# Editorial: Community series in mental-health-related stigma and discrimination: prevention, role, and management strategies, volume III

**DOI:** 10.3389/fpsyt.2025.1704050

**Published:** 2025-09-24

**Authors:** Renato de Filippis, Samer El Hayek, Mohammadreza Shalbafan

**Affiliations:** ^1^ Psychiatry Unit, Department of Health Sciences, University Magna Graecia of Catanzaro, Catanzaro, Italy; ^2^ American Center for Psychiatry and Neurology, Dubai, United Arab Emirates; ^3^ Mental Health Research Center, Psychosocial Health Research Institute, Department of Psychiatry, School of Medicine, Iran University of Medical Sciences, Tehran, Iran

**Keywords:** community psychiatry, healthcare, indirect discrimination, internalized stigma, misinformation, public health, social stigma, stigmatization

Mental-health-related stigm/a and discrimination remain critical barriers to the effective prevention, treatment, and recovery of mental illnesses (1, 2). They not only undermine help-seeking behaviors and adherence to care but also perpetuate social exclusion, inequities, and poor health outcomes (3). Addressing stigma requires a multidimensional approach that integrates prevention, clearly defined roles for healthcare professionals, communities, and policymakers, as well as evidence-based management strategies (4, 5). Understanding these dynamics is essential for developing interventions that reduce stigma, promote resilience, and foster inclusive environments supportive of mental well-being (6, 7). These issues are especially evident in the medical field, though not limited to it, and have been further exacerbated by the social and health disruptions of the COVID-19 pandemic (8–10).

In the third volume of the Community Series on *Mental-Health-Related Stigma and Discrimination: Prevention, Role, and Management Strategies*, we feature 20 new articles that examine different dimensions of stigma and discrimination in mental health, bringing together perspectives from across the globe (11, 12). This editorial highlights the central themes of these contributions and invites readers to engage more deeply with the insights offered throughout the Research Topic.

Two articles explored COVID-19-related stigma from the perspective of healthcare workers. Moradi-Gorabpasi et al. investigated psychiatrists’ perspectives on stigma related to COVID-19. They conducted a cross-sectional survey among 131 psychiatrists in Tehran using a 15-item questionnaire. Reported scores ranged from 25 to 68, with an average of 51.16 ± 8.83. Most respondents (73.3%) acknowledged the presence of stigma early in the pandemic; however, opinions diverged on whether it persists today—69.8% still perceived ongoing stigma, while others disagreed or remained neutral. Additionally, 63.3% recognized stigma associated with conditions such as AIDS and leprosy, and nearly two-thirds of these respondents also linked stigma to COVID-19. Overall, the study revealed a spectrum of views among psychiatrists, with the majority recognizing that COVID-19 stigma remains an issue.

Fahim et al. examined healthcare providers’ (HCPs) perceptions and experiences of stigma during the COVID-19 pandemic in Canada and Singapore. Using the Health Stigma and Discrimination Framework, the researchers conducted 51 qualitative interviews (23 in Canada and 28 in Singapore). Their findings revealed that stigma affected both HCPs and marginalized populations. The study illustrates how stigma intersected with race, occupation, and social marginalization in both countries, underscoring the need for targeted strategies to reduce COVID-19–related stigma among healthcare providers and vulnerable groups.

The authors of six articles explored the role of mental health stigma among HCPs. Torres and colleagues conducted a cross-sectional online survey in Portugal using the Portuguese version of the Opening Minds Stigma Scale for Healthcare Providers (OMS-HC), enrolling 292 professionals. Close contact with someone with mental illness was associated with lower social distance scores, while a personal history of mental illness corresponded to higher stigma in the disclosure/help-seeking domain (13). In the study led by Sangwan, the authors conducted a qualitative investigation across 13 primary healthcare facilities in Jodhpur, Rajasthan. A total of 25 providers—including Medical Officers, Community Health Officers, General Nurse Midwives, and Auxiliary Nurse Midwives—participated in semi-structured interviews. Four major themes emerged: (i) navigating mental healthcare in primary care, (ii) barriers and challenges to service delivery, (iii) recommendations for improving services, and (iv) strategies for training and capacity building. Overall, while providers expressed confidence in their ability to deliver mental health services, negative attitudes toward mental illness remained evident (14). Similarly, Mensah conducted a qualitative study involving semi-structured, open-ended interviews with 14 registered psychiatric nurses. The findings revealed two main forms of stigma: social stigma, which discourages many from pursuing psychiatric nursing as a career, and structural stigma, tied to government neglect and inadequate resources. Both forms of stigma were found to hinder the ability of nurses to provide effective care for individuals with mental health conditions (15). Wang et al. surveyed 628 nurses from five hospitals in Liaoning Province using a combination of online and offline methods. Willingness to work in psychiatry was found to influence stigma levels. Correlation analysis revealed significant relationships among stigma, social distance, and mental health knowledge. Mediation analysis further showed that stigma fully mediated the relationship between mental health knowledge and social distance—once stigma was accounted for, knowledge no longer had a direct effect on social distance. Similarly, the group led by Qi-Kai Wang conducted an online survey of 1,324 nonpsychiatric nurses and nursing students in Sichuan Province, China. The questionnaire collected data on demographics, personal care experience, psychiatric nursing education, and responses to the Community Attitudes toward the Mentally Ill (CAMI) scale. Results showed that higher education, longer residence in urban areas, and personal care experience were associated with increased authoritarian and social restrictiveness attitudes and decreased benevolence. Hierarchical regression analysis showed that psychiatric clinical practice was linked to reduced benevolence and lower community mental health ideology scores; however, its initial associations with authoritarianism and social restrictiveness disappeared after adjusting for confounders (16). Finally, Kılıç-Demir and Kızılpınar compared the perceptions, attitudes, and beliefs of forensic psychiatric nurses and general medicine nurses toward psychiatric patients in Turkey. The study included 46 forensic psychiatric nurses (mean age 35.46) and 58 general nurses (mean age 36.28). Results showed that forensic nurses held significantly more positive attitudes, including lower social distance, higher levels of trust, greater willingness to treat, and a reduced perception of patients as threatening. Among forensic nurses, more negative attitudes were associated with being male, single, longer working hours, and having fewer children. Additionally, insufficient knowledge about forensic psychiatry was linked to negative beliefs about mental illness and its curability. Among general nurses, having first-degree relatives receiving psychiatric treatment was correlated with perceiving patients as threatening, while age and number of siblings influenced perceptions and trust (17).

Only the study by Oblak explicitly assessed the impact of stigma on suicide risk. Drawing on stigma-related data from the 2022 and 2023 Eurobarometer surveys, along with suicide rates and socio-economic indicators across 27 European countries, the analysis found a general decline in suicide rates from 2010 to 2019, with increases observed in only four countries. Several negative associations emerged between suicide rates and stigma indicators—particularly the belief that disclosing a mental health condition could harm one’s career, which consistently predicted lower suicide rates in regression models.

Only one article specifically addressed psychiatric stigma related to infectious diseases. Gong et al. conducted a cross-sectional study involving 200 women with high-risk human papillomavirus (HR-HPV) at the Department of Gynecology, General Hospital of Northern. The findings showed that 18.5% of participants reported low stigma, 76.0% moderate stigma, and 5.5% high stigma. Stigma levels were positively correlated with rumination and negatively correlated with psychological resilience. Independent predictors of stigma included monthly income, recurrent infections, psychological resilience, and rumination, with the latter two factors together explaining 23.4% of the variance.

One article evaluated the correlations between stigma, mental health, and dermatology. Huang et al. investigated the relationships among stigma, social appearance anxiety, alexithymia, and mental health in patients with psoriasis, with particular attention to the mediating roles of social appearance anxiety and alexithymia. The findings indicated that stigma negatively impacts the psychological well-being of psoriasis patients both directly and indirectly—by increasing appearance-related anxiety and impairing emotional expression. The authors suggested that interventions targeting these mediators may help improve mental health outcomes in this population (18).

Two articles focused on stigma within the general population. Boon Tan et al. conducted a cross-sectional study investigating causal beliefs about seven mental illnesses and evaluated the revised Causal Beliefs Scale. A total of 4,195 respondents were randomly assigned vignettes depicting different conditions and asked about illness recognition, causal beliefs, and prior experience with mental illness. Compared to schizophrenia, biogenetic explanations were less endorsed for most conditions, except dementia, while personality-based explanations were strongly linked to depression with suicidality. Respondents who correctly recognized the illness were more likely to endorse biogenetic and psychosocial causes and less likely to attribute it to physical or personality factors. In a separate study, Spahlholz et al. applied a milieu approach to examine how shared social, cultural, and political orientations shape stigma toward mental illness. Using data from a 2020 nationally representative vignette-based survey of 3,042 German adults, the study found that negative stereotypes—such as beliefs that people with depression are weak-willed or that those with schizophrenia are dangerous—were more common in authoritarian-leaning milieus. In contrast, liberal-leaning groups expressed significantly less desire for social distance from individuals with depression, though these differences were less pronounced for schizophrenia (19).

Tehrani et al. and Tehrani et al. investigated the relationship between depression self-stigma and demographic factors through a cross-sectional survey with 1,075 residents of Gonabad, using proportionate stratified sampling. The findings indicated that self-stigma represents a significant barrier to seeking treatment within this community. The authors suggested that increasing mental health literacy and reducing stigma through awareness initiatives may promote help-seeking and improve access to care (20).

Only the article by Ezemenaka et al. addressed the issue of racism associated with stigma. This study examined the relationship between gendered racism, depression, and psychological distress among 200 rural Black women in the Southeastern United States. The results showed that 21.5% of participants reported depression, and 31% experienced moderate psychological distress. A higher stress appraisal of gendered racism was significantly associated with increased depression (p = 0.002). These findings highlight how gendered racial microaggressions contribute to poor mental health outcomes among rural Black women, emphasizing the need for targeted interventions and culturally responsive support.

In another study, Garmehi et al. translated and validated the Persian version of the Reported and Intended Behaviour Scale (RIBS). A total of 384 Persian-speaking adults (aged 18–60) from various Iranian cities completed the survey online. Stigmatizing behaviors were significantly associated with age, employment status, and education level. The study concluded that the Persian RIBS is a valid and reliable tool for measuring stigma toward mental illness among Iranian adults.

Two studies examined mental health-related stigma issue in student populations. Zhang and colleagues conducted a web-based cross-sectional survey of 632 students at three medical universities in China, exploring medical students’ knowledge, attitudes, and practices regarding depression management. The findings suggested that while medical students demonstrated adequate knowledge and positive attitudes toward depression, their practical skills remained limited. The authors recommended enhancing medical curricula with hands-on methods, such as role-playing and case-based learning, to bridge the gap between theoretical knowledge and effective clinical practice (21).

Hyseni Duraku et al. investigated the interplay between awareness, stigma, and help-seeking behaviors among Albanian university students in Kosovo, North Macedonia, and Albania. Using focus groups with 60 students and framed by the socio-ecological model, the study explored individual, interpersonal, organizational, and societal influences. While students demonstrated moderate awareness of mental health issues, familial and cultural stigma often inhibited open discussion and professional help-seeking. Institutional support was limited—only 20% of students felt their mental health needs were met—highlighting the lack of affordable, confidential counseling services, empathetic faculty, and supportive campus environments. Broader cultural norms further reinforced stigma, limiting access to ongoing community-based mental health care (22).

In their paper, Dá Mesquita and colleagues compared volunteer attitudes toward working with individuals with mental illness and with prisoners. A secondary qualitative analysis was conducted using transcripts from 39 semi-structured interviews with prison volunteers and two focus groups with mental health volunteers. Data were analyzed through inductive thematic analysis. Four themes emerged: (1) motivation and characteristics of volunteers, (2) roles of volunteers, (3) the impact of volunteer–beneficiary relationships, and (4) challenges faced. Both groups expressed positive attitudes, emphasized the need for specialized training, and highlighted the rewarding nature of volunteering. Alternatively, differences emerged in the skills required, the types of activities performed, and the challenges encountered across the two settings (23).

Finally, Mandangu et al. conducted a systematic review to examine how bias influences referral practices in mental health care and to identify strategies for promoting more equitable access. The review focused on professional and organizational biases across eight qualifying studies. Thematic synthesis =revealed five domains where bias affected decision-making: resource allocation, organizational procedures, clinical roles, practitioner judgment, and referral preferences. The findings suggested that implicit bias within referral systems can restrict access to patient-centered, relational therapies. The authors recommended implementing standardized referral protocols, strengthening professional training on implicit bias, and enhancing oversight to reduce disparities in mental health care.

In summary, the articles presented in this editorial underscore the urgent need for a comprehensive, multifaceted approach to addressing mental health-related stigma. This approach should integrate public awareness campaigns, targeted education, and the use of inclusive, non-stigmatizing language. The future of psychiatry must prioritize empathy, understanding, and open dialogue, thereby empowering individuals to seek support without fear of judgment or discrimination. Realizing this vision requires coordinated efforts from mental health professionals, policymakers, and communities, to dismantle stigma and promote better mental health outcomes within a more compassionate and equitable society (24–26).
